# Exploring views on alcohol consumption and digital support for alcohol reduction in UK‐based Punjabi‐Sikh men: A think aloud and interview study

**DOI:** 10.1111/dar.13172

**Published:** 2020-09-16

**Authors:** Karamjeet Taak, Jamie Brown, Olga Perski

**Affiliations:** ^1^ Research Department of Behavioural Science and Health University College London London UK

**Keywords:** hazardous alcohol consumption, digital intervention, Punjabi‐Sikh, think aloud, qualitative

## Abstract

**Introduction and Aims:**

We aimed to explore UK‐based Punjabi‐Sikh men's views on: (i) alcohol consumption within the community; (ii) available support for alcohol reduction; and (iii) an evidence‐informed alcohol reduction app.

**Design and Methods:**

Semi‐structured interviews and a think aloud method were employed. Participants (*n* = 15) were male, aged 18–27 years, identified as Punjabi‐Sikh, were hazardous or harmful drinkers (i.e. had an Alcohol Use Disorders Identification Test‐Consumption score of ≥5) and interested in using an app to reduce drinking. Interviews were audio‐recorded, transcribed verbatim and analysed with inductive thematic analysis.

**Results:**

Six themes were developed: (i) fear of drinking to cope; (ii) clash between religious and cultural norms (i.e. an internal conflict between important values); (iii) stigmatisation of mental health issues and lack of knowledge as barriers to help seeking; (iv) perceived usefulness of goal setting, monitoring and feedback (i.e. beliefs about the utility of the app's components for reducing drinking); (v) concerns about accessibility of the app within the Punjabi‐Sikh community; and (vi) desire for human support for continued app engagement.

**Discussion and Conclusions:**

Among UK‐based, Punjabi‐Sikh men, clashing religious and cultural norms give rise to internal conflict about drinking. Stigmatisation of mental health issues and lack of knowledge of available support leads to reduced help seeking. Respondents believed an evidence‐informed alcohol reduction app could be useful, but were concerned about accessibility within the wider community and wanted an element of human support. The potential for a combination of digital and face‐to‐face support should be explored.

## Introduction

Hazardous and harmful alcohol consumption is estimated to be high among UK‐based Punjabi‐Sikh men at 16–41% [1, 2]. In the United Kingdom (UK), alcohol‐related hospital admissions are markedly higher in Indian compared with white men [3]. Heavy drinking is more common among Punjabi‐Sikh men than women [4], with alcohol consumption considered a defining feature of masculinity [5, 6]. In contrast, abstinence from alcohol is typically expected in women of South Asian origin [4]. Although effective face‐to‐face support for alcohol reduction is available in the UK, opportunistic, brief interventions are rarely delivered in primary care [7, 8]. In addition, individuals from Black and minority ethnic groups tend to be under‐represented in UK alcohol support services [4], which is at least partly attributable to stigma of admitting to ‘problem drinking’ [9], lack of knowledge of available services and language barriers [10]. For example, many Punjabi‐Sikh families with a member who drinks excessively express worry about others finding out, suggesting that heavy drinking is stigmatised within the community [11]. This may be further explained by research indicating that Sikh families experience pressure to conform to religious values [12], with Sikh men reporting that they tend to drink alone or at home, as opposed to socially or publicly, thus, suggesting that alcohol consumption is kept private [1]. This fear of stigma may act to deter Punjabi‐Sikh men from seeking health‐care professional support to reduce drinking [10].

A recent rapid evidence review highlighted several barriers to accessing support for alcohol reduction in Black and minority ethnic groups [10]; however, little is known about the views of Punjabi‐Sikh men on digital support for alcohol reduction. As alcohol services may be avoided in the hope of keeping one's drinking private, digital interventions may be a promising means of support in this population. Digital interventions are becoming increasingly popular, with high rates of smartphone ownership reported across the UK population [13]. Recent systematic reviews have found that interactive digital interventions for alcohol reduction are effective compared with static or wait‐list controls [14, 15]. Digital interventions exist in several forms, including computer programs or websites that are to be used alongside face‐to‐face support from a health‐care professional (i.e. ‘blended’ or ‘guided’ interventions) and stand‐alone computer programs or smartphone apps which require the user to interact with a smart device on their own.

A qualitative approach is particularly suited for generating deep insight into social and cultural influences on alcohol consumption and perceptions of digital support for alcohol reduction in hard‐to‐reach groups [16]. Using in‐depth interviews and a think aloud method, this study aimed to explore the views of UK‐based, Punjabi‐Sikh men on: (i) alcohol consumption within the community; (ii) available support for alcohol reduction; and (iii) an evidence‐informed alcohol reduction app.

## Method

### Study design

Semi‐structured interviews were used to explore participants' views on alcohol consumption and available support for alcohol reduction. A think aloud method, which involves asking participants to verbalise their thoughts, impressions and feelings while completing a given task [17], was used to explore participants' impressions of the content and graphical user interface of an evidence‐informed alcohol reduction app, *Drink Less*. The *Drink Less* app was selected as it was developed using theory and best available evidence, and there is tentative evidence of short‐term effectiveness from a factorial screening trial [18]. *Drink Less* was developed for the general population of adult, excessive drinkers in the UK [18, 19]. The app's content was informed by the COM‐B model [20], which posits that the interaction of capability (physical and psychological), opportunity (physical and social) and motivation (reflective and automatic) leads to behaviour such as hazardous or harmful drinking. A standalone paper details exactly how the COM‐B model and evidence informed the app's development [19]. The app version used in the current study (v.1.10) was centred around a goal setting module with five additional modules: (i) normative feedback; (ii) cognitive bias re‐training; (iii) self‐monitoring and feedback; (iv) action planning; and (v) identity change [19].

University College London's Departmental Ethics Committee granted ethical permission (CEHP/2016/556). Personal identifiers were removed from the data, which were stored securely. Participants provided informed consent prior to taking part in the study.

An interpretivist approach was used, which focuses on building a subjective view of the social world from the participant's perspective and recognises the active role of the researcher in both the elicitation and interpretation of the data [21]. The interpretivist paradigm stands in contrast to the positivist paradigm, which assumes that qualitative research can shed light on an objective reality “out there” [22].

### Participants

Participants were eligible if they were: (i) aged 18 years or over; (ii) male; (iii) identified as Punjabi‐Sikh; (iv) lived in or near London and were able to come into University College London for an interview (this criterion was revised towards the end of the study period to ensure an adequate sample size was achieved; participants who met all other criteria were offered to take part remotely via Skype); (v) scored 5 or above on the Alcohol Use Disorders Identification Test‐Consumption (AUDIT‐C) scale [23, 24]; (vi) owned an iPhone with internet access that was capable of running apps (as the *Drink Less* app is currently only available for iOS); and (vii) were interested in using a smartphone app to reduce their alcohol consumption.

Participants were recruited via adverts placed on social media platforms (i.e. Facebook, Twitter, LinkedIn) and mental health charities for UK‐based Punjabi‐Sikhs (e.g. ‘Taraki’) and snowball sampling, whereby participants were asked to refer friends or family members. Participants were recruited in batches of three until theoretical saturation was judged to have occurred (i.e. when no new themes were identified). As is common in qualitative research, intermittent data analysis was conducted after each batch of three participants to determine whether more participants were needed [25].

A total of 15 male participants aged 18–27 years took part. Participants had AUDIT‐C scores of 5–9, indicating hazardous or harmful alcohol consumption. Weekly alcohol consumption estimated from the typical frequency and volume questions of the AUDIT‐C ranged from 4 to 18 standard UK units.

### Procedure

Participants who expressed an interest in taking part were sent a link to an online screening survey, which included the information sheet and consent form. Eligible participants were invited to take part in an interview, conducted in a private space at University College London or via Skype. No one else was present in the interviews except for the first author and the participant. Sessions lasted between 40 and 60 min (*M* = 45.9, *SD* = 8.15). Due to restrictions on time and travel, 5 of 15 participants were interviewed via Skype. Participants were encouraged to be open and honest and were reassured that any information disclosed was confidential. A topic guide was developed to explore participants' views on alcohol consumption and support for alcohol reduction (see Appendix S1, Supporting Information). After every three participants, questions were revised to maximise the quality of responses.

Participants were instructed on how to think aloud. They were asked to complete a practice task (i.e. thinking aloud while changing the ringtone on their phone). Once they felt comfortable, participants were asked to download the *Drink Less* app from the Apple App Store and were prompted to complete four tasks: (i) complete the onboarding procedure and view the normative feedback; (ii) record some past drinks in the calendar; (iii) set a weekly alcohol reduction goal of their choice; and (iv) play the ‘Yes Please, No Thanks’ game (i.e. part of the cognitive bias re‐training module). When participants fell silent, prompts were used (e.g. “What are you thinking now?”). Participants were then asked to summarise their overall impression of the *Drink Less* app, and whether they would consider using the app again in the future (see Appendix S1). Upon completion of the interview, participants were entered into a prize draw to win a £20 shopping voucher.

### Data analysis

Sessions were audio‐recorded, transcribed verbatim and analysed using inductive thematic analysis by the first and last author [26]. Initial codes were generated and higher‐order themes that captured participants' underlying ideas, beliefs and conceptualisations were developed. Before producing the report, themes and quotations were reviewed for coherency and validity.

External validation aims to ensure agreement between the researcher's interpretation of the data and participants' accounts [27]. A random subsample of three participants (20%), selected via www.random.org, was contacted via e‐mail and asked to review the results following the development of initial themes. Participants were asked to comment on whether they felt that their views were well represented and the extent to which they agreed with the interpretation of their quotations. All three participants responded and indicated that they agreed with the authors' interpretations.

Reflexivity refers to the observation that the researcher's attributes and perspective are likely to have an influence on participants' accounts [28]. The researcher (female, young adult, identifies as Punjabi‐Sikh) felt that she established a good rapport with the participants. A discursive style was used to generate more extensive data. The researcher did not feel that the gender difference had a negative impact on participants' responses but believed that they were more likely to open up because of the age similarity and because the researcher was a community member. Particularly when referring to the ‘older generation’, participants appeared to assume that the researcher could relate to this (e.g. “I don't think it's that much of a concern for *our* age group”). Similarly, participants used terms that are likely to be understood only by community members or those with sufficient knowledge of Sikhism (e.g. ‘amritdhari’, ‘gurdwara’).

## Results

Six themes were generated. Given the structure of the topic guide, which first explored views on alcohol consumption within the Punjabi‐Sikh community and subsequently asked participants to carry out the think aloud tasks with the *Drink Less* app, the data and themes naturally fell into two broad subsections. Three themes were developed in relation to respondents' views on alcohol consumption within the Punjabi‐Sikh community and available support for alcohol reduction: (i) fear of drinking to cope; (ii) clash between religious and cultural norms; and (iii) stigmatisation of mental health issues and lack of knowledge as barriers to help seeking. Three themes were developed in relation to respondents' views on the *Drink Less* app: (i) perceived usefulness of goal setting, monitoring and feedback; (ii) concerns about the accessibility of the app within the Punjabi‐Sikh community; and (iii) desire for human support for continued app engagement.

### Views on excessive alcohol consumption and available support for alcohol reduction

#### Fear of drinking to cope

Participants' views of drinking within the community were strongly shaped by having observed older relatives drinking heavily throughout their formative years. Participants emphasised that alcohol was a common form of escapism, but only for the older generation, and were quick to highlight ways in which their own relationship with alcohol differed from that of the older generation. Witnessing older relatives ‘turn to alcohol’ in response to stress or using alcohol as a ‘pain killer’, and how this in turn led to physical health problems or impacted negatively on family relations, led participants to avoid using alcohol as a means to cope. Instead, they emphasised that they tended to drink in social situations ‘to have a good time’. As such, alcohol was described as a social lubricant and a way to relax or mitigate boredom.

*‘There's family issues, marriage issues, financial issues. You can't just be like, let's just take a week out and go on holiday, because you have responsibilities. Alcohol is a short‐term escape*’. P6, age 19

‘*To be honest I wouldn't really turn to it if I'm down or stressed or whatever, because I don't want that to become my life. I've seen others do that and where it can lead to, so I only really drink if I'm partying or with friends to have a good time*’. P11, age 21

The majority of respondents did not believe that their own drinking patterns were ‘problematic’, mainly because they did not negatively impact on important others, and so did not express a strong intention to reduce their drinking. Despite recognising that they sometimes drank heavily, participants viewed their own drinking as safe and controlled, unlike other heavy drinkers or ‘alcoholics’, who would benefit from cutting down (i.e. the act of ‘othering’; [29]).

‘*I do get really drunk, but I do know like whilst I am under the influence of alcohol, I don't let it affect people around me. I stay within my own limits and I know how to look after myself, but there are people where it does have implications for people around them. That's when it gets serious—when your addictions start affecting other people*’. P1, age 23

As such, participants were not concerned about the health or social consequences of their own drinking and appeared to endorse the belief that one's drinking is harmful only if it negatively affects family members, relatives or friends.

#### Clash between religious and cultural norms

Respondents described being introduced to alcohol by older relatives at an early age (i.e. <18 years), and that they expected alcohol to form part of social gatherings with other Punjabi‐Sikhs. Alcohol was considered so engrained within the community that those who declined a drink at social events were viewed as the ‘odd one out’.

*‘When there's a function, there has to be alcohol there. If there's not, then people are like: “What?! How is there not alcohol there?!”’* P6, age 19

‘*At this point, it's so prevalent amongst the social functions and home life it would be a tall order to remove alcohol*’. P15, age 23

The anticipated surprise described by participants when imagining a social event without alcohol can be interpreted to suggest that alcohol plays a key role in Punjabi culture, particularly at celebratory events such as birthdays or weddings, which stands in stark contrast to religious norms within Sikhism.

Participants described feeling negatively influenced by Punjabi popular culture (e.g. music videos, lyrics), particularly through its glorification of drinking and perpetuation of masculinity stereotypes, often leading to drinking contests.

‘*I think it's quite bad, to be honest. Bad because it's glorified. Like if you drink more, you're a bigger guy, which is bad*’. P2, age 22

Participants explained how Sikhism prohibits alcohol consumption and how this, in combination with Punjabi popular culture and social norms within the peer group, led to a veritable clash between important values. For some participants, this internal conflict meant that they actively made the decision to distance themselves from Sikhism.

‘*A reason why I stopped wearing a turban was because I was kind of indulging in alcoholism and for me, if I was to follow Sikhi, I would want to do it properly*’. P1, age 23

The decision for some participants to distance themselves from Sikhism due to feelings of guilt about not adhering to the religious tenets implies that there is strong social pressure within Punjabi culture for men to participate in heavy drinking.

#### Stigmatisation of mental health issues and lack of knowledge as barriers to help seeking

Participants described heavy drinking and mental health issues as being stigmatised within the community, thus preventing people from seeking support to drink less. Personal difficulties tended to be kept within the immediate family to avoid embarrassment or shame, as mental health issues were viewed as a ‘weakness’, particularly among the older generation. Some participants described the community in general and gurdwaras (a place of assembly and worship) in particular as being ‘judgmental’ towards those who drink to excess.

‘*I don't think support is seen as an option within our community. There seems to be a lack of awareness. There's also that fear of shame if someone finds out you have an actual problem*’. P9, age 20

Participants believed that helpseeking behaviours differed between generations due to differences in education and awareness of mental health issues. Older relatives were described as ‘stubborn’ and ‘stuck in their ways’, unwilling to acknowledge that they might benefit from health‐care professional support to reduce drinking. However, when asked where they would turn if they felt that they needed support to reduce their alcohol consumption, the majority of participants stated that they did not know where to seek support. Two participants mentioned being aware of community support groups held in Birmingham gurdwaras, but not in London.

‘*To be honest, I wouldn't know where to seek help. But if I had a problem, I would look at my social group and see how they influence me. I know support groups exist and the NHS* [National Health Service] *does stuff. But I don't know in too much detail*’. P5, age 20

Although participants perceived themselves as more open to discussing mental health issues compared with the older generation, they expressed a lack of awareness of where to find available support for alcohol reduction.

### *Views on the* Drink Less *app*

#### Perceived usefulness of goal setting, monitoring and feedback

When exploring the *Drink Less* app, participants expressed a strong liking for the personalised weekly goals. They further thought that the drinking calendar would be useful for monitoring their drinking and tracking progress, fostering motivation to continue to work towards achieving their goals. However, some participants commented that they did not see themselves coming back to log their drinks on a regular basis, particularly after heavy drinking sessions.

‘*I feel like when you write things down, you're accountable to seeing them through. I think the tracking and showing you the improvement—and feeling satisfactory when you see yourself doing well—I can see that working*’. P2, age 22

‘*I'm not sure how much use you'd get out of this. Just because it would be difficult to keep going back to an app to record drinks and stuff*’. P12, age 23

This highlights the difficulty in encouraging users to engage with digital support over time; even when a component is perceived as useful, users often struggle to return to the digital support over time due to perceived burden.

Despite valuing some of the app's components, most participants described the game as ‘childish’ or ‘boring’ and questioned its ability to affect behaviour change due to presenting sugary, non‐alcoholic drinks as viable alternatives to alcoholic drinks.

‘*But this is basically telling me to drink loads of coke then instead of drinking alcohol, so what does it really solve?*’ P2, age 22

This may be interpreted to suggest that participants did not distinguish between the health consequences of the heavy consumption of sugary and alcoholic drinks, which were both seen as unhealthy, thus further explaining their low motivation to reduce drinking despite meeting criteria for hazardous drinking. In addition, the majority of participants expressed surprise or shock when viewing the normative feedback which compared their drinking with others in the same gender and age group. Instead of prompting reflection on their own patterns of drinking, this led some participants to question the accuracy of the normative feedback.

*‘Wow. That doesn't seem accurate. This is bollocks!*’ P6, age 19

#### Concerns about accessibility of the app within the Punjabi‐Sikh community

Most participants stated that the app had a visually pleasing user interface, which they considered easy to navigate.

‘*I liked the visual for the alcohol comparison, the arrow on the meter, that's handy to see and gives a good representation of my drinking*. […] *The colours are good too*’. P12, age 23

However, many participants thought that they would lose interest in the app after minimal use. For some participants, the app's interface was described as evoking similar reactions to that of visiting the doctor. Participants also raised accessibility concerns: they thought that older Punjabi‐Sikh community members may experience language barriers and struggle with the amount of text within the app. Half of participants stated that the wording of messages within the app would benefit from being simplified.

*‘The questions are a bit long winded and I'm dyslexic so it's taking me a bit of time to read the questions. I also feel like if it were used in the Punjabi community, it would need more language options*’. P5, age 20

#### Desire for human support for continued app engagement

Some participants believed that the app would be most effective for the Punjabi‐Sikh community if it were used alongside guidance and support from a peer group or health‐care professional. Participants believed that committing to the use of an app to reduce drinking would require discipline; having someone to report back to was considered important for encouraging continued app engagement.

‘*I can see it being used alongside a different form of therapy. If you went to group meetings where you were to share your results on the app with other people. I think it could be accepted in the community, alongside a different form of therapy and a professional body*’. P7, age 27

‘*I think it could be effective within group settings because then you're held accountable if you have to answer to someone. It forces you to take it more seriously*’. P15, age 27

Echoing concerns about not being able or willing to return to the app to log their drinks in the calendar, the expressed desire for human support can be interpreted to suggest that participants anticipated feeling more motivated to reduce drinking if there was a social or health‐care professional element to the digital support.

When asked if they would use the app again after the study, 5 of 15 participants said ‘yes’, 6 of 15 said ‘no’ and the remaining 4 said ‘yes’, but only after improvements had been made or after having had the opportunity to further explore the app in their own time.

## Discussion

### Principal findings

This study used a qualitative approach to explore the views of UK‐based, Punjabi‐Sikh men on alcohol consumption within the community and an evidence‐informed alcohol reduction app. Participants expressed a strong desire for their relationship with alcohol to be different from that of the older generation, who tended to drink to cope with stressors. Participants experienced a clash between religious and cultural norms, which sometimes resulted in the distancing of oneself from Sikhism (but not from Punjabi culture). Help‐seeking behaviours tended to be avoided due to stigmatisation of mental health issues and a lack of awareness of where to find support. Participants believed that an evidence‐informed alcohol reduction app would be useful, particularly the goal setting, self‐monitoring and feedback components, but expressed concerns about accessibility within the Punjabi‐Sikh community and a desire for adjunct human support.

Many participants expressed the belief that their own drinking was not ‘problematic’ in a way that warranted help seeking and that they tended to drink in social situations to ‘have fun’ but pointed out examples of alcohol‐related behaviours that they believed would warrant support. This has previously been described as the act of ‘othering’—a social phenomenon whereby excessive drinkers perceive themselves as separate from ‘alcoholics’, who are thought to ‘drink until they have no control’ [29].

Our findings support and extend those in the extant literature. For example, the differences in drinking practices and motives between the older and younger generations echo findings in the general alcohol literature, with younger adults tending to drink due to social motives (rather than drinking to cope) [30] and coping motives being more common in older drinkers [31], who also tend to drink more frequently [32]. In addition, participants believed that the evidence‐informed *Drink Less* app would be useful, particularly components consistent with Control Theory (i.e. goal setting, self‐monitoring and feedback) [33]. In addition, some participants believed the app would be most useful alongside human support, as this may increase users' commitment to the change process and app engagement, thought to be necessary for effective self‐management. The role of human support in digital interventions has previously been emphasised, with ‘blended’ interventions (i.e. those combining digital and health‐care professional or peer support) generating greater levels of user engagement [34, 35, 36] and in turn, increased intervention effectiveness [15].

### Strengths and limitations

To the best of our knowledge, this was the first study to explore the views of UK‐based Punjabi‐Sikh men on digital support for alcohol reduction. Participants were young, recruited on the basis of being interested in using an app to reduce their drinking, and although participants were categorised as hazardous drinkers, they were at the lighter end of the spectrum with weekly consumption ranging from 4 to 18 standard UK units. It is likely that these factors influenced participants' attitudes towards drinking in general and the selected app in particular. For pragmatic purposes, the geographic sample frame was limited to participants who lived in London. However, experiences of available support for alcohol reduction, which can influence motivation to reduce drinking, may differ across geographic regions. For example, two participants mentioned being aware of community support for alcohol reduction in Birmingham (but not in London). Hence, future research should consider nationwide recruitment, as this may yield further insight. In addition, an amendment to the study protocol to ensure that a sufficient number of participants was recruited meant that a third of participants were interviewed via Skype. This might have influenced the ability of the researcher to establish rapport with participants: for participants whose interviews took place face‐to‐face, the conversational nature of their entry into the building and journey to the interview room may have encouraged participants to open up to the researcher. In contrast, Skype interviews began almost immediately following a brief, informal introduction. On the other hand, previous research examining differences between interviews conducted face‐to‐face versus over the phone has found that the latter is more conducive to participants opening up due to reduced social desirability and increased feelings of anonymity [37]. It is hence plausible that the blending of face‐to‐face and Skype interviews may have led to differences in participants' accounts of their own and others' drinking. However, we found no evidence of a difference in depth and breadth [37] across interview modalities, with theoretical saturation judged to have occurred after 15 interviews due to common patterns identified across interviews.

### Avenues for future research

As participants expressed a desire for human support alongside digital interventions and concerns about the accessibility of the *Drink Less* app to the Punjabi‐Sikh community (e.g. due to a lack of language options), future research should use principles from co‐design to explore different means of providing support in the UK‐based Punjabi‐Sikh community [38]. Although some community members are interested in receiving digital support, others may not be interested in a standalone app but may benefit from face‐to‐face support from a health‐care professional or peer group. Researchers and practitioners involved in the design of digital support for this population should consider the incorporation of different language options, short intervention messages and content designed specifically to mitigate the act of ‘othering’ (e.g. normative feedback on alcohol consumption with cultural/religious affiliation added as a key dimension alongside age and gender). These design recommendations are subject to future research; however, research with other minority ethnic groups speaks to the importance of cultural adaptation to promote user engagement with digital alcohol interventions [39] and to overcome existing health and social disparities [40].

## Conclusion

Among UK‐based, Punjabi‐Sikh men, clashing religious and cultural norms give rise to internal conflict about alcohol consumption. Stigmatisation of mental health issues within the community leads to reduced help seeking. Respondents believed an evidence‐informed alcohol reduction app could be useful but were concerned about accessibility and wanted an element of human support.

## Conflict of interest

KT and OP report no conflicts of interest. JB has received unrestricted research funding from Pfizer to study smoking cessation.

## Supporting information

**AppendixS1**. Interview topic guide.
