# Supplementary material for: Exploring views on alcohol consumption and digital support for alcohol reduction in UK‐based Punjabi‐Sikh men: A think aloud and interview study
Source: Drug Alcohol Rev. 2020 Sep 16;40(2):231–8. doi: 10.1111/dar.13172 (PMC8436744; doi:10.1111/dar.13172)
Supplement: Supplementary file 1 — AppendixS1. Interview topic guide. [file DAR-40-231-s001.docx]

**Interview topic guide**

1. When did you first start consuming alcohol?
2. What settings do you usually drink in?
3. How has your drinking changed since you first started?
4. Do you feel like you drink more in certain situations? If so, which ones?
   1. Prompt - Are there any particular moods or feelings that influence your drinking?
5. What are your views on alcohol consumption within the Punjabi-Sikh community?
   1. Prompt - Do you think it is seen as a permissible/accepted behaviour?
6. If you were looking to reduce your alcohol consumption, where would you seek support? Why?
   1. Prompt - For example, support within the NHS/treatment centres/helplines?
7. Shortly, I will ask you to download the Drink Less app. What features or functionalities do you expect an alcohol reduction app to include?

“Before we finish, I would like you to elaborate on a few things you mentioned during the think aloud tasks.”

1. You mentioned that you thought that [app feature/design aspect] was […]. Can you tell me a bit more about that?
2. What is your overall impression of the *Drink Less* app?
3. Do you think you might use the app again in the future? Why/why not?
4. Would you recommend the app to someone else? Why/why not?
